# Genetically encoded photocrosslinkers locate the high-affinity binding site of antidepressant drugs in the human serotonin transporter

**DOI:** 10.1038/ncomms11261

**Published:** 2016-04-19

**Authors:** Hafsteinn Rannversson, Jacob Andersen, Lena Sørensen, Benny Bang-Andersen, Minyoung Park, Thomas Huber, Thomas P. Sakmar, Kristian Strømgaard

**Affiliations:** 1 Department of Drug Design and Pharmacology, Center for Biopharmaceuticals, University of Copenhagen, Jagtvej 162, DK-2100 Copenhagen, Denmark; 2 Laboratory of Chemical Biology & Signal Transduction, The Rockefeller University, 1230 York Avenue, New York, New York 10065, USA; 3 Lundbeck Research Denmark, H. Lundbeck A/S, Ottiliavej 9, DK-2500 Valby, Denmark; 4 Department of Neurobiology, Care Sciences and Society, Center for Alzheimer Research, Karolinska Institutet, Huddinge 141 57, Sweden

## Abstract

Despite the well-established role of the human serotonin transporter (hSERT) in the treatment of depression, the molecular details of antidepressant drug binding are still not fully understood. Here we utilize amber codon suppression in a membrane-bound transporter protein to encode photocrosslinking unnatural amino acids (UAAs) into 75 different positions in hSERT. UAAs are incorporated with high specificity, and functionally active transporters have similar transport properties and pharmacological profiles compared with wild-type transporters. We employ ultraviolet-induced crosslinking with *p*-azido-L-phenylalanine (azF) at selected positions in hSERT to map the binding site of imipramine, a prototypical tricyclic antidepressant, and vortioxetine, a novel multimodal antidepressant. We find that the two antidepressants crosslink with azF incorporated at different positions within the central substrate-binding site of hSERT, while no crosslinking is observed at the vestibular-binding site. Taken together, our data provide direct evidence for defining the high-affinity antidepressant binding site in hSERT.

## Abstract (web summary)

Molecular details of how antidepressant drugs bind to the human serotonin transporter are not currently clear. Here, the authors introduce photo-cross-linkers into the protein and map the binding site of several antidepressants.

## Graphical abstract

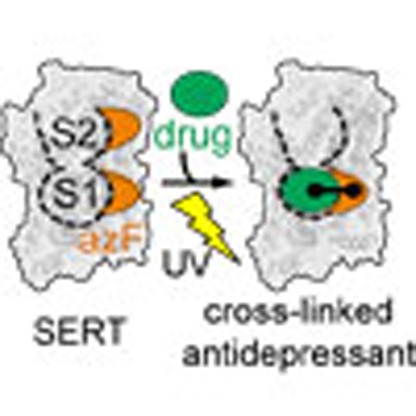

Communication between the cells of the nervous system is mediated by chemical neurotransmitters that are released from vesicles in the presynaptic neuron, diffuse across the synapse and bind to their target receptor proteins on the postsynaptic neuron. Synaptic signal transmission is regulated by the reuptake of released neurotransmitter from the synapse into the neurons by integral membrane-bound neurotransmitter transporters^1^. The serotonin transporter (SERT) is a member of the solute carrier 6 family of transporters, which also includes the transporters for the neurotransmitters norepinephrine, dopamine, glycine and γ-aminobutyric acid^2^. SERT controls the magnitude and duration of serotonergic neurotransmission by facilitating sodium-, potassium- and chloride-dependent reuptake of released serotonin (5-hydroxytryptamine; 5-HT) against its concentration gradient^2^. Pharmacological modulation of SERT transport activity influences a variety of neurophysiological processes, and inhibitors of SERT have been extensively used in the treatment of psychiatric diseases, such as depression and anxiety, with >30 drugs in current clinical use^3^.

X-ray crystal structures of bacterial and invertebrate homologues of SERT, including the bacterial amino acid transporters MhsT and LeuT^4^,^5^,^6^, and the *Drosophila melanogaster* dopamine transporter (dDAT)^7^,^8^,^9^,^10^, have proven to be excellent structural templates for their mammalian counterparts. These structures have suggested inhibitor binding at two distinct sites: the central substrate-binding site (designated the S1 site) and a vestibular site (designated the S2 site), which is located on the extracellular side of the S1 site (Fig. 1a). While mutational studies of SERT^11^,^12^,^13^,^14^,^15^,^16^, in combination with crystal structures of dDAT^7^,^8^,^9^,^10^, have suggested that antidepressants bind within the S1 site, selective serotonin reuptake inhibitors (SSRIs) and tricyclic antidepressants (TCAs) bind in the S2 site of LeuT^17^,^18^,^19^,^20^. The S2 site was first described as a secondary substrate-binding site in LeuT^21^, but has also recently been suggested to harbour an allosteric inhibitor-binding site in human transporters^22^. The precise role of the S2 site in inhibitor binding is therefore under debate, and studies that can unequivocally address this issue are much needed.

Site-specific incorporation of light-sensitive unnatural amino acids (UAAs) into proteins is a powerful approach to investigate molecular mechanisms as well as biological function with high spatial and temporal resolution. We therefore decided to employ amber codon suppression technology to introduce photocrosslinking UAAs into SERT. The methodology relies on the recognition of a nonsense codon, such as the low-abundance amber (UAG) stop codon, in the gene of interest using an orthogonal transfer RNA (tRNA) that is aminoacylated with a UAA by an aminoacyl tRNA synthetase (aaRS)^23^,^24^,^25^,^26^. Specifically, the photocrosslinking amino acids *p*-azido-L-phenylalanine (azF) and *p*-benzoyl-L-phenylalanine (BzF; Fig. 1b), which crosslink on ultraviolet radiation, have been used to gain detailed insight into protein–protein interactions^27^,^28^,^29^,^30^. Importantly, azF and BzF have also been incorporated into integral membrane protein drug targets, such as G protein-coupled receptors^31^,^32^,^33^,^34^,^35^,^36^,^37^,^38^ and ionotropic receptors^39^,^40^,^41^, to understand receptor function and to map ligand-binding sites. However, the technology has so far not been employed in studies of neurotransmitter transporters.

We demonstrate that the photocrosslinking UAAs azF and BzF can be introduced into human SERT (hSERT) with high efficiency and specificity. We further find that imipramine, a prototypical TCA, and vortioxetine, a novel multimodal antidepressant, irreversibly crosslink to azF located at different positions in the S1-binding site of hSERT, while no crosslinking is observed when azF is inserted into the S2-binding site. Our results prove the feasibility of introducing genetically encoded photoreactive UAAs into a neurotransmitter transporter and also provide direct evidence for the location of the high-affinity inhibitor-binding site in hSERT.

## Results

### hSERT is amenable to the incorporation of UAAs

**Figure 1:**
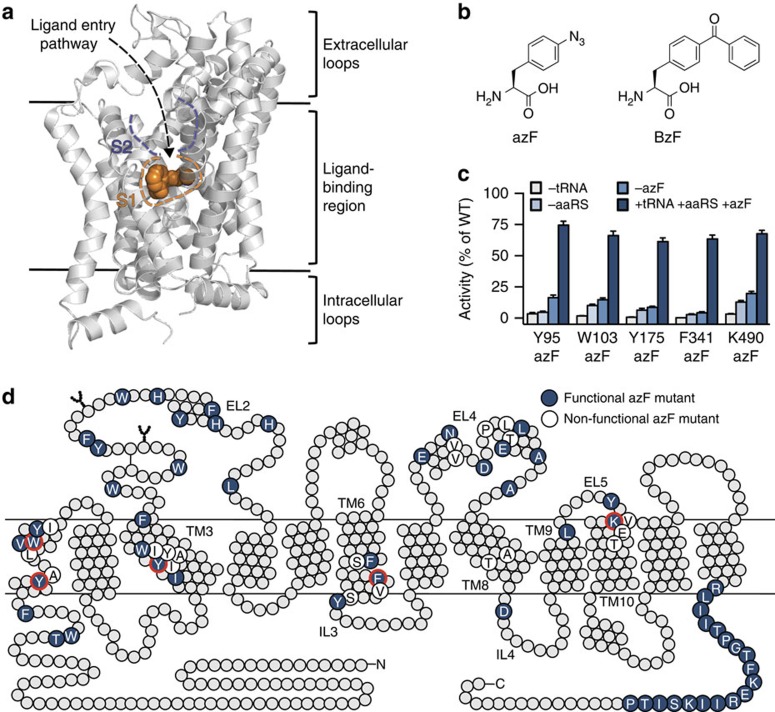
Introduction of photocrosslinking UAAs into hSERT. (**a**) X-ray crystal structure of dDAT with nortriptyline bound (PDB ID 4M48). The location of the S1- and S2-binding sites are illustrated with orange and purple dashed lines, respectively. TM10 and TM11 have been removed for clarity. (**b**) Chemical structures of azF and BzF. (**c**) Specificity of azF incorporation. The [^3^H]5-HT uptake activity of five azF mutants (Y95azF, W103azF, Y175azF, F341azF and K490azF) where one of the transfection components (suppressor tRNA, aaRS or azF) was omitted. Data are represented as mean±s.e.m. from three independent experiments each performed in triplicate. (**d**) Two-dimensional schematic representation of hSERT showing the location of the 75 positions that were mutated to azF or BzF. Blue circles indicate positions where azF introduction resulted in a functionally active transporter (defined as >5% functional activity relative to WT hSERT activity), while white circles indicate positions where introduction of azF abolished activity (see Supplementary Table 1 and Supplementary Fig. 1 for transport activity of BzF mutants). Circles highlighted with a red line designate the positions examined in **c**. TM, transmembrane.

To explore the feasibility of incorporating the photocrosslinking amino acids azF and BzF (Fig. 1b) into hSERT in a comprehensive and systematic manner, we co-transfected HEK293T cells with plasmids carrying the genes of hSERT containing an amber codon at a desired position along with an orthogonal suppressor tRNA/aaRS pair for azF or BzF. The cells were cultured in media containing either azF or BzF, and subsequently the translational efficiency and fidelity were assessed in a functional [^3^H]5-HT uptake assay. We probed 75 positions in hSERT, representing different side-chain structures and spatial locations in the protein (Fig. 1d), where azF or BzF was introduced through amber codon suppression mutagenesis, resulting in 150 hSERT mutants.

In the majority of the tested positions, azF or BzF could be genetically encoded into hSERT and led to a functionally active transporter protein (uptake activity of >5% compared with wild-type (WT) hSERT). Specifically, of the 75 positions examined, the functional activity was retained in 55 (73%) of the azF mutants and in 47 (63%) of the BzF mutants (Fig. 1d, Supplementary Results; Supplementary Fig. 1; Supplementary Table 1). Our comprehensive data set allowed us to evaluate putative correlations between the nature of the native amino acid, as well as spatial location, and efficiency of incorporation of azF and BzF (Supplementary Table 2). Generally, mutations of native aromatic residues to either azF or BzF were better tolerated compared with mutations of non-aromatic residues (96% versus 65% for azF; 78% versus 56% for BzF). The incorporation of BzF at the transmembrane segments was tolerated at 20% of the positions tested, while 44% of the transmembrane mutants retained functionality for azF incorporation. The loop and termini regions were highly amenable to both azF and BzF incorporation with only a single terminal BzF mutant not being functional (Supplementary Table 2). Furthermore, the specificity and fidelity of azF incorporation were determined at five different positions of the transporter (Y95, W103, Y175, F341 and K490; Fig. 1d), by measuring the activity in cells where one of the transfection components (tRNA, aaRS or azF) was omitted (Fig. 1c). Negligible functional activity (<10%) was observed when suppressor tRNA or aaRS was absent from the transfections. In the absence of azF, generally a markedly reduced activity was observed. However, for K490 in particular, we did observe some read-through in the absence of azF, which is due to imperfect fidelity for azF by the engineered aaRS/tRNA pair^35^.

### Introduction of azF into the S1 and S2 sites in hSERT

**Figure 2:**
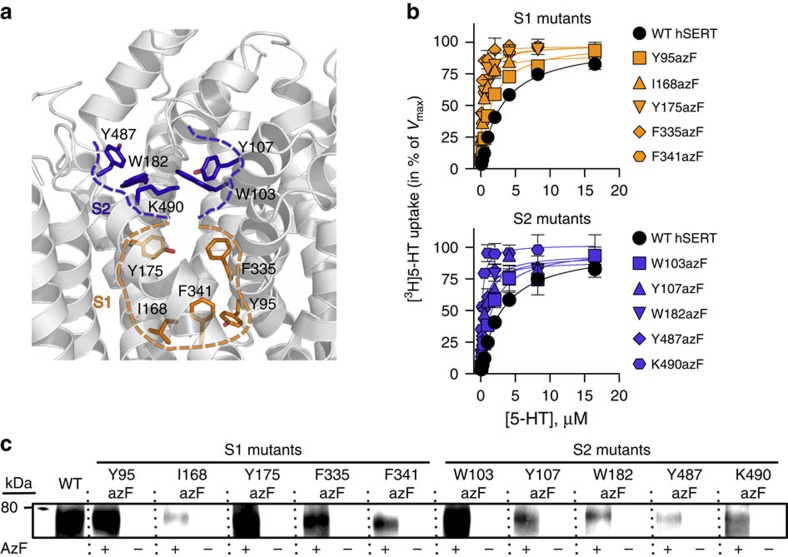
Characterization of hSERT containing photocrosslinking UAAs around the S1- and S2-binding sites. (**a**) Homology model of hSERT showing the residues in the S1- and S2-binding sites that were mutated to azF. Residues located within the S1 and S2 sites are shown in orange and purple, respectively. (**b**) [^3^H]5-HT uptake saturation curves (normalized to the maximal rate of transport, *V*_max_) in HEK293T cells expressing WT hSERT and the five S1 azF mutants (orange symbols) or the five S2 azF mutants (purple symbols), illustrating the effects of the azF mutations on the uptake kinetics of the transporter (see Supplementary Table 3 for *K*_m_ and *V*_max_ values). Data points are represented as mean±s.e.m. from a representative experiment with triplicate determinations. (**c**) Immunoblot analysis of lysates from HEK293T cells expressing WT hSERT or azF mutants, using a mAb against a C-terminal 1D4 epitope fused to hSERT. Using an epitope at the C terminus of the transporter, only full-length protein is detected in the immunoblot analysis. Cells were grown either in the presence or absence of azF. For the azF mutants, no full-length protein was detected in the absence of azF, illustrating that translation stopped when the amber stop codon was reached.

After establishing the amber codon suppression technique for incorporation of photocrosslinking UAAs into hSERT, we wanted to explore if UAAs could be introduced in regions of hSERT relevant for mapping of drug-binding sites. The initial screen of 75 residues was used to identify functionally active mutants surrounding the putative inhibitor-binding sites (S1 and S2). Due to a better incorporation tolerance and generally higher activities of azF mutants compared with BzF mutants, we chose to focus on azF mutants. We selected five mutants surrounding the S1-binding site (Y95azF, I168azF, Y175azF, F335azF and F341azF) and five mutants surrounding the S2-binding site (W103azF, Y107azF, W182azF, Y487azF and K490azF), based on spatial orientation and distance from the proposed binding sites in a homology model of hSERT^42^ (Fig. 2a). Initially, we analysed the expression of the 10 azF mutants by immunoblotting. The immunoblot analyses were performed on HEK293T cells expressing azF mutants of hSERT, using Rho1D4 monoclonal antibody (mAb) against a C-terminal 1D4 epitope fused to hSERT to ensure that only full-length protein would be detected. A clear band was detected for WT hSERT and each of the 10 azF mutants at 75–80 kDa, corresponding to mature glycosylated hSERT^43^. Consistent with the lower activity of the 10 azF mutants compared with WT hSERT (Supplementary Table 1), the intensities of the bands corresponding to the azF mutants were weaker than the band from WT hSERT in all cases, indicating a lower expression level of the mutants. Importantly, no band corresponding to full-length hSERT was detected in any of the samples from cells grown in the absence of azF (Fig. 2c).

To investigate whether the incorporation of azF affected the transport kinetics, cells expressing azF mutants of hSERT were incubated with increasing concentrations of the substrate (5-HT) and the maximum rate of transport (*V*_max_) and the substrate concentration needed to achieve half maximum rate of transport (*K*_m_) were determined. Introduction of azF into the S1 or S2 site led to decreased *V*_max_ values, whereas the *K*_m_ values of the mutants were in the same range as WT hSERT, with the exception of F335azF, F341azF and K490azF, where the *K*_m_ values decreased by 6- to 13-fold (Fig. 2b; Supplementary Table 3). Thus, despite a lower transport activity caused by lower cell surface expression (Fig. 2c), the 10 azF mutants retained sufficient uptake activity for robust and reliable measurements.

### Mapping of drug-binding sites using amber codon suppression

**Figure 3:**
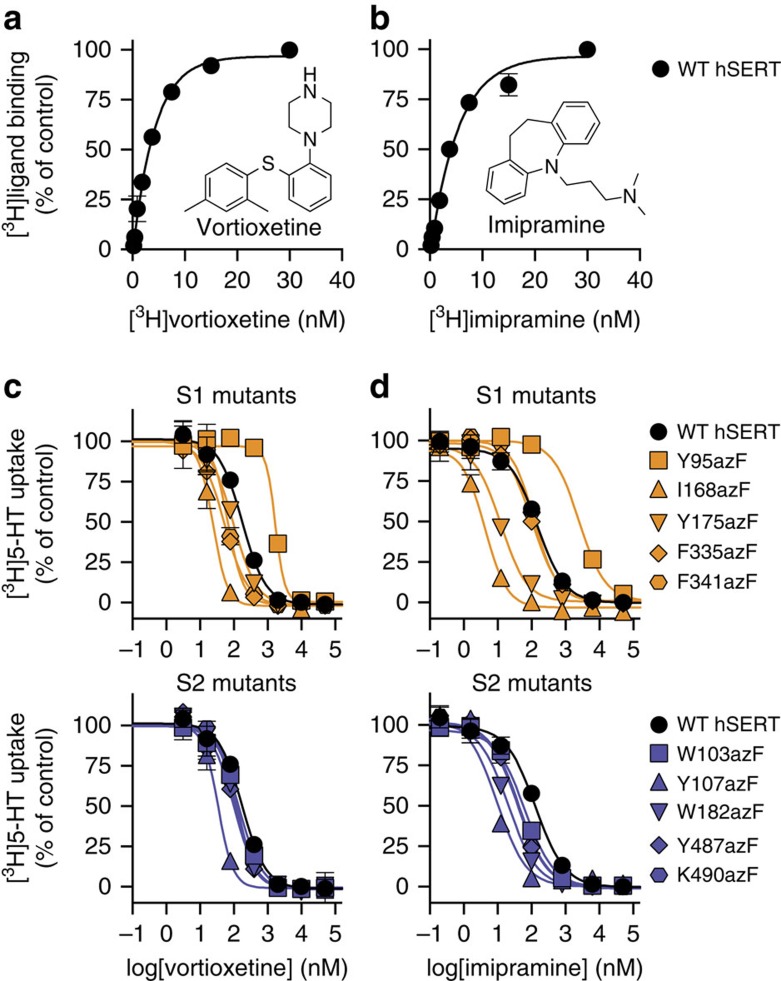
Binding of vortioxetine and imipramine to hSERT. (**a**) Saturation binding curve for [^3^H]vortioxetine binding to HEK293T membranes expressing WT hSERT. Vortioxetine binds to WT hSERT with a *K*_d_ of 6.3±0.7 nM (mean±s.e.m. from 11 independent experiments each performed in triplicate). (**b**) Saturation binding curve for [^3^H]imipramine binding to HEK293T membranes expressing WT hSERT. Imipramine binds to WT hSERT with a *K*_d_ of 7.2±0.8 nM (mean±s.e.m. from six independent experiments each performed in triplicate). (**c**,**d**) Inhibition of [^3^H]5-HT uptake by vortioxetine (**c**) or imipramine (**d**) in HEK293T cells expressing WT hSERT, one of the five S1-binding site mutants (orange symbols) or one of the five S2-binding site mutants (purple symbols), illustrating the effect of the mutations on the potency of vortioxetine and imipramine. *K*_i_ values for vortioxetine and imipramine on WT hSERT and the 10 azF mutants are included in Supplementary Table 3. Each data point represents mean±s.e.m. from a representative experiment carried out in triplicate. A C-terminal 1D4 epitope was fused to WT hSERT and 10 azF mutants. Importantly, this epitope did not affect the binding affinity of vortioxetine or imipramine (Supplementary Fig. 2).

Next, the potential of the amber codon suppression technique for mapping of the drug-binding sites in hSERT was investigated. Specifically, we examined whether antidepressant drugs could undergo ultraviolet-induced crosslinking to any of the 10 S1/S2 azF mutants of hSERT, and thereby provide unambiguous evidence that the drug binds in close proximity (<3 Å)^31^ to the given azF side chain. We chose two pharmacologically and structurally different antidepressant drugs for these studies: the classical TCA drug imipramine and vortioxetine, a newly marketed multimodal antidepressant drug, which in addition to potent inhibition of hSERT (Fig. 3a,b) also binds to and modulates the activity of different serotonergic receptors^44^,^45^. To determine the binding affinity of the two drugs for WT hSERT, we performed saturation binding assays using [^3^H]labelled versions of imipramine and vortioxetine, and found that WT hSERT binds vortioxetine and imipramine with dissociation constants (*K*_d_) of 6.3±0.7 and 7.2±0.8 nM, respectively (Fig. 3a,b; Supplementary Fig. 2).

To examine whether the 10 S1/S2 azF mutants affected the potency of the two drugs, the inhibition constants (*K*_i_) of vortioxetine and imipramine for WT hSERT and the 10 azF mutants were determined in a [^3^H]5-HT uptake inhibition assay (Fig. 3c,d). Generally, the S2-binding site mutations had modest effects on the potency of imipramine and vortioxetine, except for the Y107azF mutant, which was 5-fold and 12-fold more sensitive to vortioxetine and imipramine, respectively (Fig. 3c,d; Supplementary Table 3). More noticeable changes were seen for the S1-binding site mutants. The most pronounced effects were seen for I168azF, which was significantly more sensitive to both vortioxetine and imipramine (10-fold and 33-fold, respectively), whereas Y95azF decreased the potency of both compounds (6-fold and 21-fold, respectively; Fig. 3c,d; Supplementary Table 3). Our mutational data indicated that the inhibitors bind to the central substrate site in hSERT, and is thus consistent with the fact that imipramine bind competitively with 5-HT^46^. Furthermore, we found that vortioxetine does not change the *V*_max_ value but significantly increases the *K*_m_ value of 5-HT transport (Supplementary Fig. 3), strongly suggesting that vortioxetine also binds competitively with 5-HT in the central substrate site of hSERT.

**Figure 4:**
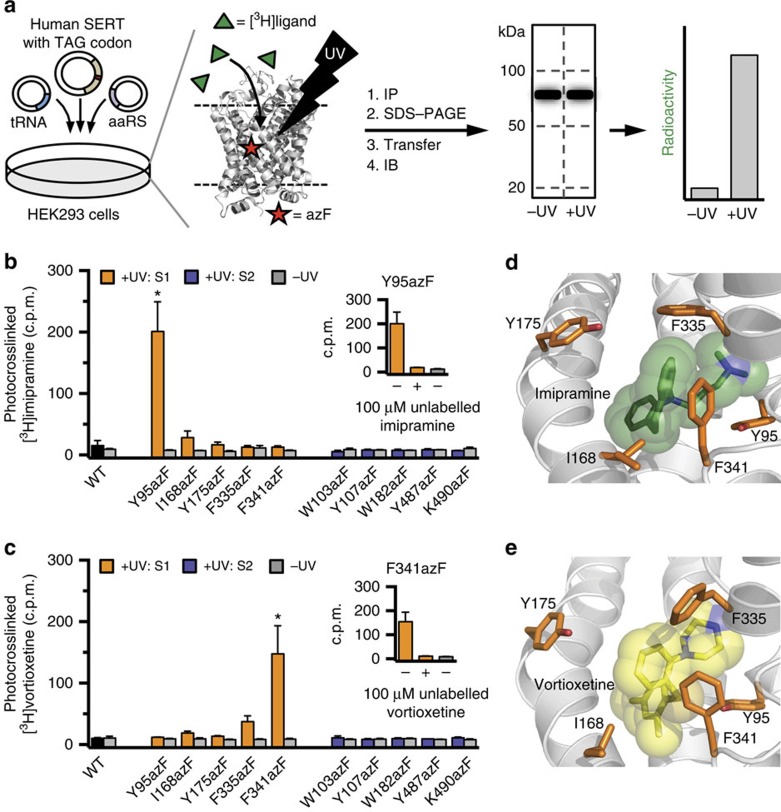
Crosslinking studies and binding site determination of vortioxetine and imipramine. (**a**) Schematic illustration outlining the photocrosslinking experiments. Briefly, HEK293T cells expressing WT or azF mutants of hSERT were incubated with 100 nM of [^3^H]vortioxetine or [^3^H]imipramine and exposed to ultraviolet (UV) light. Cells were lysed, the transporter immunopurified (IP) and run on an SDS–PAGE gel, before transferring to PVDF membrane for immunoblotting (IB). Each lane of the membrane was cut into three segments (20–50, 50–100 and >100 kDa, respectively) and the radioactivity of each segment was quantified. As control, non-UV-exposed samples were in all cases assayed in parallel. (**b**,**c**) Results from the photocrosslinking experiments between hSERT azF mutants, and [^3^H]imipramine (**b**) and [^3^H]vortioxetine (**c**). The bars represent the radioactivity signal expressed as counts per minute (c.p.m.) detected in PVDF membrane segments corresponding to 50–100 kDa that contain hSERT. For the 20–50 and >100 kDa segments, the radioactivity signal was in all cases indistinguishable between UV-exposed and control samples (Supplementary Fig. 4). Asterisks (*) denotes significant difference in crosslinking signal compared with non-UV-exposed samples (*P*<0.05; two-way analysis of variance with Sidak multiple comparisons test). Inserts show that crosslink of [^3^H]imipramine to Y95azF (**b**) and [^3^H]vortioxetine to F341azF (**c**) was fully outcompeted in the presence of 100 μM unlabelled inhibitor added before UV exposure. Data are represented as mean±s.e.m. from three to seven independent experiments. (**d**,**e**) Docking models of imipramine^51^ (**d**) and vortioxetine^42^ (**e**) binding in the S1 site in hSERT. Imipramine is shown in green (**d**), vortioxetine is shown in yellow (**e**) and the five S1 residues that were mutated to azF are shown as orange sticks.

Since all 10 S1/S2 mutants bound both the inhibitors, we investigated their ability to form covalent crosslinks to [^3^H]labelled drugs. In these experiments, WT hSERT and the 10 azF mutants were transfected into the same batch of HEK293T cells and the 10 mutants and WT hSERT were assayed in parallel with each drug. Specifically, HEK293T cells expressing either WT or azF mutants of hSERT were incubated with 100 nM of either [^3^H]vortioxetine or [^3^H]imipramine at 4 °C for 2 h to reach binding equilibrium (Fig. 4a). After incubation, cells expressing a given azF mutant were divided into two batches, one of which was exposed to ultraviolet light to induce the chemical crosslinking reaction, while the other was used as a control to assess the level of non-ultraviolet-induced crosslink. After cell lysis and immunopurification of the transporters using Rho1D4 mAb, the samples were separated by SDS–polyacrylamide gel (SDS-PAGE) electrophoresis and protein transferred onto a polyvinylidene fluoride (PVDF) membrane for immunoblotting. Each sample lane of the PVDF membrane was subsequently divided into three segments containing protein with the molecular weights of 20–50, 50–100 or >100 kDa (Fig. 4a). Consistent with the initial immunoblot analyses (Fig. 2c), the bands corresponding to hSERT were located in the 50–100-kDa segments (Supplementary Fig. 6). Finally, each membrane piece was placed in a scintillation vial containing scintillation fluid to quantify the radioactivity of each sample (Fig. 4a), where a radioactivity signal indicated irreversible chemical crosslink between the azF hSERT mutant and the [^3^H]labelled drug.

Importantly, no radioactivity signal was observed in WT hSERT after incubation of either [^3^H]vortioxetine or [^3^H]imipramine, showing that the ultraviolet-induced crosslinking did not occur when azF was not genetically encoded (Fig. 4b,c; Supplementary Fig. 4). Examination of the radioactivity patterns for the two inhibitors at the 10 azF mutants revealed significant insight into the specificity of crosslinking. For [^3^H]imipramine, there was no difference between the radioactivity signals from the ultraviolet-exposed samples and the control samples at any of the S2-binding site mutants, showing that [^3^H]imipramine did not crosslink to any of these mutants. In contrast, the radioactivity signal was significantly higher in the ultraviolet-induced sample of the S1 mutant Y95azF compared with the control sample, showing that [^3^H]imipramine was covalently attached to the Y95azF mutant upon ultraviolet radiation (Fig. 4b). The signal was detected in the 50–100 kDa segment of the membrane, in agreement with the size of mature glycosylated SERT, while no radioactivity was detected in the 20–50 or >100 kDa membrane segments (Supplementary Fig. 4). Similarly, no radioactivity signal was detected in any of the control samples that were not exposed to ultraviolet light, demonstrating that crosslinking of [^3^H]imipramine was dependent on ultraviolet exposure (Fig. 4b).

For [^3^H]vortioxetine, the radioactivity signals for ultraviolet-induced and control samples were also indistinguishable for the five S2 mutants (Fig. 4c). Interestingly, a different pattern was observed with the S1 mutants for vortioxetine compared with imipramine. A significant ultraviolet-dependent radioactivity signal was detected at F341azF when incubated with [^3^H]vortioxetine, while no signal was detected at Y95azF, which elicited a signal for imipramine (Fig. 4c). The signal at F341azF was exclusively detected in the 50–100 kDa membrane segment (Supplementary Fig. 4). Furthermore, a full displacement of the radioactivity signal at Y95azF and F341azF for imipramine and vortioxetine, respectively, was observed when 100 μM of unlabelled inhibitor was added before ultraviolet exposure, further demonstrating the specificity of the signal (Fig. 4b,c).

From these results, we conclude that the radioactivity signals detected at Y95azF and F341azF for imipramine and vortioxetine, respectively, were a result of direct ultraviolet-induced crosslink between the [^3^H]labelled drugs and azF inserted into hSERT, thereby providing direct evidence for inhibitor binding in the S1-binding site of hSERT.

## Discussion

Considering the role of hSERT as a principal drug target in the treatment of affective disorders, obtaining a better understanding of drug binding is of great clinical importance. Intensive efforts to understand the molecular basis of antidepressant binding to hSERT have mainly relied on site-directed mutagenesis and cysteine accessibility studies. However, these studies do not provide direct experimental information about inhibitor binding, and it has been difficult to assign specific inhibitor-binding functionalities unambiguously based on these mutational studies due to potential indirect and long-range allosteric effects on protein structure. Photoaffinity analogues of tropane-based inhibitors, such as cocaine, and SSRIs, including escitalopram, have been developed to define drug–protein interactions at the molecular level in DAT and SERT, respectively^47^,^48^,^49^,^50^. However, although photoaffinity ligands can provide a defined anchor point for ligand orientation within a binding pocket, addition of the relatively large photoreactive functional group to a drug likely affects its binding mode. X-ray crystal structures of dDAT co-crystallized with antidepressants suggest that the high-affinity inhibitor-binding site in hSERT overlaps the central S1 site^7^,^8^,^10^. In contrast, studies on hSERT have suggested that inhibitor binding is not limited to the S1 site^22^, which is also supported by X-ray crystal structures of LeuT complexed with TCAs and SSRIs in the S2 region^17^,^18^,^19^. Since no X-ray crystal structures of hSERT in complex with antidepressants have yet been obtained, many important uncertainties remain regarding inhibitor binding at hSERT.

In the present study, we demonstrate the use of amber codon suppression in a membrane transporter protein and report direct evidence for the location of the high-affinity antidepressant-binding site in hSERT. Specifically, we show that two structurally and pharmacologically different antidepressant drugs, imipramine and vortioxetine, can be covalently attached to azF when this photocrosslinking UAA is inserted into the central S1 site in hSERT, thereby directly showing that the binding site for vortioxetine and imipramine overlaps the central substrate-binding site. In contrast, no ultraviolet-induced photocrosslinking to imipramine or vortioxetine was observed when azF was inserted into the vestibular S2 site of hSERT (Fig. 4b,c). Noteworthy, since we were aiming at mapping the high-affinity binding site for vortioxetine and imipramine, we employed a concentration of 100 nM of the drugs in the photocrosslinking experiments. Under these conditions, a low-affinity binding site may not be occupied, and the lack of covalent photocrosslinking signal in S2, cannot rule out the presence of a secondary, allosteric inhibitor-binding site in this vestibular site.

The S1 site in hSERT is a narrow and constrained area that is located in the core of the transporter protein (Fig. 1a). Previously established binding models for imipramine^51^ and vortioxetine^42^ in hSERT suggest that both the drugs primarily interact through hydrophobic interactions to S1 residues in addition to formation of a salt bridge between an aspartate residue (Asp98) and a positively charged nitrogen atom in the drugs. Furthermore, Tyr95 has been suggested as a key interaction site for both imipramine and vortioxetine^16^,^42^,^51^,^52^, and we indeed found that the Y95azF mutant decreased the potency of the two drugs (Fig. 3c,d). We also found that inserting azF into position 168 (I168azF) in the bottom of the S1 site increased the potency of both inhibitors (Fig. 3c,d). Similarly, mutation of Ile168 has previously been shown to increase the potency of vortioxetine and other inhibitors, such as fluoxetine^42^,^53^. Ile168 is assumed to be located close to the aromatic moieties of vortioxetine and imipramine (Fig. 4d,e), and the increased inhibitor binding could be mediated by additional hydrophobic interactions. Interestingly, we found that azF mutants in the S2 site generally induced an increased potency of both imipramine and vortioxetine (Fig. 3c,d; Supplementary Table 3). Most prominent was the effect of Y107azF, which induced 5- and 12-fold increase in potency of vortioxetine and imipramine, respectively. Since the inhibitors have to permeate through the S2 site to reach the central S1 site (Fig. 1a), the increased potency could be induced by altering the kinetics of ligand binding (that is, by inducing a faster association to or a slower dissociation from the S1 site) or the S2 mutations could alter the conformational equilibrium of the transporter, and thereby allosterically affect ligand binding in S1. It is noteworthy that some of the azF mutants induce 6- to 13-fold decreased *K*_m_ value as found for F335azF, F341azF and K490azF (Fig. 2b; Supplementary Table 3). This is in line with previous studies showing that other mutations in the same positions such as F335W, F341Y and K490F induce 6- to 16-fold decreased *K*_m_ values in hSERT^13^,^14^, and thus substantiates that these three residues hold an important role for substrate recognition and/or are important determinants for the conformational equilibrium. Specifically, F335 forms a conserved hydrophobic lid together with Y176 close to the top of the S1 site, and movement of the aromatic side chain of F335 has been proposed to be an important trigger for the shift between outward-open and inward-open conformations^10^. The decreased *K*_m_ value in F335azF could thus be induced by altering the conformational equilibrium of hSERT.

Interestingly, all of the five positions in which azF was introduced into the S1-binding pocket (Y95, I168, Y175, F335 and F341) are located in close proximity of the proposed binding sites of imipramine^51^ and vortioxetine^42^, and thereby represent potential candidates for undergoing ultraviolet-induced photocrosslinking to the drug molecules (Fig. 4d,e; Supplementary Fig. 5). Importantly, primary conclusions can only be made in cases where a positive crosslinking signal is observed, and a positive crosslink is only possible if there is a close physical interaction between the engineered transporter and the ligand in the presence of ultraviolet light. We found that vortioxetine and imipramine were only covalently attached to a single, but different, azF mutant within the S1 site (Y95azF for imipramine; F341azF for vortioxetine). For imipramine, it is notable that Y95azF displayed the highest degree of photocrosslinking even when this azF mutant decreased the potency of the drug (Fig. 3d). The decreased potency could result from the added bulk of the azide group relative to the phenolic hydroxyl, but the significant photocrosslinking to Y95azF unequivocally show that imipramine bind in close proximity of Y95 within the S1 site as proposed previously^16^,^52^. Upon ultraviolet illumination, azF forms the highly reactive nitrone that can undergo nucleophile addition resulting in covalent crosslinking^54^. The amino groups of vortioxetine and imipramine can serve as nucleophiles in crosslink reactions with azF, and these nucleophilic moieties thus represent obvious crosslinking attachment sites on the drugs. Docking models of vortioxetine and imipramine in hSERT (Fig. 4d,e; Supplementary Fig. 5) show that Y95 is located closer (∼1 Å) to the dimethylamino moiety of imipramine, compared with the piperazine ring of vortioxetine. In contrast, F341 is located >1 Å closer to the piperazine ring of vortioxetine compared with the dimethylamino group of imipramine. Hence, the proposed docking models of vortioxetine and imipramine in hSERT are consistent with the selective photocrosslinking to Y95azF and F341azF that we found for imipramine and vortioxetine, respectively.

In summary, we have shown direct evidence of antidepressant binding at hSERT using a targeted photocrosslinking strategy with genetically encoded UAAs. The amber codon suppression technology applied to neurotransmitter transporters has allowed site-specific crosslinking of two unmodified antidepressant drugs in the S1-binding site of hSERT at distinct juxtaposed UAA side chains. We believe that this general method will be important in future studies that will provide additional functional and mechanistic insights into this important class of membrane proteins, including molecular level insights into transporter structure and function.

## Methods

### Materials

DMEM, fetal bovine serum, trypsin and penicillin–streptomycin was purchased from Life Technologies. DNA restriction enzymes and T4 DNA ligase enzyme were from New England Biolabs. Cell culture dishes were from Sarstedt AG & Co, and white 96-well plates were from Nunc. [^3^H]5-HT (27–28 Ci mmol^−1^), [^3^H]imipramine (41 Ci mmol^−1^), MicroScint-0 and -20 scintillation fluid and 96-well glass fibre filter plates were obtained from PerkinElmer Life Sciences. [^3^H]vortioxetine (69 Ci mmol^−1^) was purchased from Ubichem Pharma Services (Hungary). azF and BzF were from Chem-Impex International, Inc. Rho1D4 mAb was obtained from The National Cell Culture Center.

### Molecular biology

Plasmids containing the suppressor tRNA, azF aaRS or BzF aaRS^35^,^36^ and the plasmid pcDNA3.1 hSERT^13^ were used for transfections. Unless otherwise noted, all experiments were conducted on hSERT constructs C-terminally fused to the 1D4 epitope. The 1D4 epitope, TETSQVAPA, was added to hSERT by replacing the coding sequence for WT hSERT with a PCR-generated insert containing the coding sequence for hSERT carrying the C-terminal 1D4 epitope. PCR primers were purchased from MWG Eurofins with restriction sites for XhoI and XbaI for the 5′ and 3′ primers, respectively. Generation of amber codon (TAG) mutations in SERT-1D4 was performed by site-directed mutagenesis using the QuickChange mutagenesis kit (Stratagene). 1D4 tag insertion and amber codon mutations were verified by DNA sequencing of the entire coding domain sequence (GATC Biotech).

### Cell culturing and expression of SERT azF and BzF mutants

HEK293T cells (American Type Culture Collection, Manassas, VA) were cultured in DMEM media with 10% fetal bovine serum, 100 U ml^−1^ penicillin and 100 μg ml^−1^ streptomycin at 37 °C in a humidified 5% CO_2_ environment^16^. Cells were transfected at 60–70% confluency using TransIT LT-1 (Mirus) or Lipofectamine 2000 (Life Technologies) transfection reagent, following the protocol supplied by the manufacturer. The plasmid DNA ratio hSERT/suppressor tRNA/aaRS was 1/1/0.1 by mass. For transfections with lipofectamine in a 10-cm plate, 8.25 μg of hSERT amber codon mutant DNA was used with 52 μl of lipofectamine 2000. For transfections with TransIT LT-1 in a 96-well plate, 4.8 μg of hSERT amber codon mutant DNA was used with 30 μl of TransIT LT-1. For transfection in other types of plates, amounts were scaled according to surface area. Transfected cells were grown in media supplemented with 0.5 mM of either azF or BzF.

### Uptake assays

Uptake assays were performed 40–48 h post transfection in 96-well plates. Cells were washed twice with PBSCM buffer (in mM: NaCl, 137; KCl, 2.7; Na_2_HPO_4_, 4.3; KH_2_PO_4_, 1.4; CaCl_2_, 0.1; MgCl_2_, 0.5) on an ELx50 automated microplate strip washer (BioTek Instruments) and left with phosphate-buffered saline containing CaCl_2_ and MgCl_2_ (PBSCM) for 20 min before uptake experiments. For uptake activity studies, cells were incubated with 50 μl PBSCM containing 50 nM [^3^H]5-HT. For the determination of *K*_m_ and *V*_max_ values, cells were incubated with 50 μl of PBSCM containing increasing concentrations of [^3^H]5-HT (diluted 1:10 or 1:20 with cold 5-HT). In inhibition studies (half-maximal inhibitory concentration (IC_50_) determinations), cells were preincubated in 40 μl of PBSCM with increasing concentrations of inhibitor for 30 min before the addition of 20 μl of PBSCM containing [^3^H]5-HT giving a final substrate concentration of 200 nM. In all experiments, uptake was allowed to proceed for 5 min at 20 °C and was terminated by washing three times with PBSCM (ELx50 automated microplate strip washer). The amount of accumulated radiolabelled substrate was determined by solubilizing cells in scintillation fluid (MicroScint-20), followed by counting of plates in a Packard TopCounter. All control experiments on WT hSERT were carried out in the background of the co-transfected orthogonal azF or BzF tRNA/aaRS pair, with cells incubated in the presence of azF or BzF. Non-specific uptake was determined in parallel as uptake in non-transfected HEK293T cells. Uptake assays were carried out in triplicate and repeated at least three times unless otherwise noted.

### Cell membrane preparation and radioligand-binding assay

Membranes were prepared from HEK293T cells transiently expressing hSERT growing on 15-cm tissue culture plates. At 40–48 h post transfection, cells were suspended in PBS and centrifuged at low speed (700*g*) for 5 min. The cell pellet was resuspended in cold H_2_O and frozen at −20 °C for 1 h. The suspension was then thawed on ice and subjected to 10–15 passages through a 21-Ga needle to disrupt cells. The homogenate was centrifuged at 18,000*g* at 4 °C for 30 min, the supernatant fraction was aspirated and the pellet was resuspended in PBSCM. Protein concentration was determined by the BCA method using Pierce bicinchoninic acid (BCA) Protein Assay (ThermoFischer). Membranes were used directly for binding assays or stored at −80 °C until use. For saturation binding studies, increasing concentrations of [^3^H]imipramine or [^3^H]vortioxetine were incubated with 5 or 15 μg total membrane protein per sample in a total volume of 150 μl PBSCM. Binding proceeded for 2 h on ice with gentle rocking. Subsequently, membranes were transferred to 96-well glass fibre filter plates preincubated with 0.1% polyethyleneimine (30 μl per well) using a Packard Bell cell harvester and washed four times with water. Non-specific binding was determined in parallel with membranes from non-transfected HEK293T cells. Filter plates were dried and soaked in scintillation fluid (MicroScint-0), followed by counting in a Packard Topcounter. Saturation binding assays were carried out in triplicate and repeated at least three times.

### Immunoaffinity purification

HEK293T cells growing in 15-cm culture dishes and transiently expressing 1D4-tagged hSERT mutants were suspended in PBS 40–48 h post-transfection, pelleted by centrifugation (700*g*, 5 min) and solubilized (2 h, 4 °C) in solubilization buffer (50 mM Tris-HCl, pH 7.5, 145 mM NaCl, 5 mM EDTA and 1% Triton X-100 or IGEPAL CA-630, supplemented with protease inhibitors (cOmplete, Roche)). After centrifugation (18,000*g*, 30 min), the supernatant fraction was incubated overnight at 4 °C with Dynabeads Protein G (Life Technologies) bound to Rho1D4 mAb or with sepharose 2B beads conjugated to Rho1D4 mAb. Beads were washed five times with solubilization buffer and Dynabeads Protein G bound samples were eluted for 10 min at 70 °C with LDS loading buffer (Life Technologies), while sepharose bead samples were eluted twice for 20 min at 60 °C with LDS loading buffer.

### Gel electrophoresis and immunoblot analysis

Protein samples were subjected to SDS–PAGE on a 4–12% Bis-Tris gel (Life Technologies) and transferred to a PVDF membrane. Detection was performed using the SNAP i.d. Protein Detection System (Millipore). PBS-T (0.1% Tween 20 in PBS) was used as washing buffer and 0.2% non-fat dried milk in PBS-T was used as blocking reagent. Blots were incubated 10 min with Rho1D4 mAb (1:1,000 dilution of a 1 mg ml^−1^ stock), followed by 10-min incubation with a horseradish peroxidase-conjugated anti-mouse Ab (1:20,000 dilution of a 1 mg ml^−1^ stock) (Promega). Blots were developed using enhanced chemoluminescence detection reagents (GE Healthcare) and visualized using a DNR MicroChemi System (DNR Bio-Imaging Systems, Ltd). For photocrosslinking studies, the immunoblot analysis was performed using TBS-T (0.1% Tween 20 in 20 mM TBS) as washing buffer and 5% non-fat dried milk in TBS-T as blocking buffer. Blots were incubated with primary Rho1D4 mAb (1:3000 in 5% blocking buffer) overnight at 4 °C. After five washes with TBS-T, the PVDF membrane was incubated 1 h at room temperature with secondary anti-mouse Ab conjugated with horseradish peroxidase (1:20,000 in 5% blocking buffer). The blot was developed using Supersignal West Pico Chemiluminescent Substrate (Thermo Scientific) before being exposed to HyBlot CL auto-radiography film (Denville Scientific Inc.). See Supplementary Fig. 6 for representative western blot of azF mutants of hSERT.

### Photocrosslinking of [^3^H]vortioxetine and [^3^H]imipramine

At 40–48 h post transfection, HEK293T cells growing in 10-cm culture dishes and transiently expressing 1D4 epitope-tagged WT or azF hSERT mutants were pelleted by centrifugation (700*g*, 5 min) and resuspended in Hank's Buffered Salt Solution (pH 7.5) containing 20 mM HEPES, 0.2% bovine serum albumin and 100 nM of either [^3^H]vortioxetine or [^3^H]imipramine. Cell suspensions were incubated for 2 h at 4 °C on a nutator, followed by exposure to a Maxima ML-3500 S UV-A light (Spectronics Corporation) in a 4 °C cold room on ice for 15 min. After ultraviolet light exposure, cells were pelleted (1,500*g*, 10 min), the supernatant fraction was removed and the pellet was stored at −20 °C until further analysis. For quantification of crosslinked [^3^H]vortioxetine and [^3^H]imipramine, cell pellets were solubilised in 1% Triton X-100 in 20 mM Tris-HCl, pH 7.5, containing protease inhibitors for 1 h at 4 °C on a nutator. After solubilization, the lysate was centrifuged (20,000*g*, 10 min) and the supernatant was immunopurified as described above. The purified protein was analysed by SDS–PAGE and immunoblot. The PVDF membrane was then cut between each lane to separate samples, and each lane was further cut into three segments covering 20–50, 50–100 and >100 kDa. Each membrane segment was soaked in scintillation fluid (Ecoscint A, National diagnostics) and counted in a Tri Carb 2900 TR liquid scintillation counter (Perkin-Elmer). Non-ultraviolet-exposed samples were in all cases assayed in parallel experiments.

### Data analysis

All data analyses were performed using Prism 6 software (GraphPad, Inc.). For determination of the Michaelis–Menten constant (*K*_m_), data from *K*_m_ assays were fitted by equation (1),


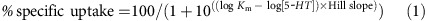


For determination of IC_50_ values, data from [^3^H]5-HT uptake inhibition assays were fitted by equation (2),


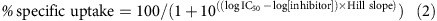


The *K*_i_ for inhibitors were calculated from IC_50_ values using the Cheng–Prusoff equation (equation (3))^55^,


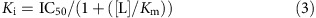


where [L] is the concentration of [^3^H]5-HT. *K*i values were compared using Student's *t*-test unless otherwise indicated. For determination of *K*_d_ values, data from saturation binding assays were fitted by equation (4),


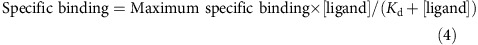


## Additional information

**How to cite this article:** Rannversson, H. *et al*. Genetically encoded photocrosslinkers locate the high-affinity binding site of antidepressant drugs in the human serotonin transporter. *Nat. Commun.* 7:11261 doi: 10.1038/ncomms11261 (2016).

## Supplementary Material

Supplementary Information Supplementary Figures 1-6, Supplementary Tables 1-3 and Supplementary References,

## References

[b1] Torres G. E., Gainetdinov R. R. & Caron M. G. Plasma membrane monoamine transporters: structure, regulation and function. Nat. Rev. Neurosci. 4, 13–25 (2003). 12511858 10.1038/nrn1008

[b2] Kristensen A. S. . SLC6 neurotransmitter transporters: structure, function, and regulation. Pharmacol. Rev. 63, 585–640 (2011). 21752877 10.1124/pr.108.000869

[b3] Andersen J., Kristensen A. S., Bang-Andersen B. & Strømgaard K. Recent advances in the understanding of the interaction of antidepressant drugs with serotonin and norepinephrine transporters. Chem. Commun. 3677–3692, doi:10.1039/b903035m (2009). 10.1039/b903035m 19557250

[b4] Yamashita A., Singh S. K., Kawate T., Jin Y. & Gouaux E. Crystal structure of a bacterial homologue of Na^+^/Cl^−^-dependent neurotransmitter transporters. Nature 437, 215–223 (2005). 16041361 10.1038/nature03978

[b5] Krishnamurthy H. & Gouaux E. X-ray structures of LeuT in substrate-free outward-open and apo inward-open states. Nature 481, 469–474 (2012). 22230955 10.1038/nature10737 PMC3306218

[b6] Malinauskaite L. . A mechanism for intracellular release of Na^+^ by neurotransmitter/sodium symporters. Nat. Struct. Mol. Biol. 21, 1006–1012 (2014). 25282149 10.1038/nsmb.2894 PMC4346222

[b7] Penmatsa A., Wang K. H. & Gouaux E. X-ray structure of dopamine transporter elucidates antidepressant mechanism. Nature 503, 85–90 (2013). 24037379 10.1038/nature12533 PMC3904663

[b8] Penmatsa A., Wang K. H. & Gouaux E. X-ray structures of Drosophila dopamine transporter in complex with nisoxetine and reboxetine. Nat. Struct. Mol. Biol. 22, 506–508 (2015). 25961798 10.1038/nsmb.3029 PMC4608549

[b9] Wang H. . Structural basis for action by diverse antidepressants on biogenic amine transporters. Nature 503, 141–145 (2013). 24121440 10.1038/nature12648 PMC3904662

[b10] Wang K. H., Penmatsa A. & Gouaux E. Neurotransmitter and psychostimulant recognition by the dopamine transporter. Nature 521, 322–327 (2015). 25970245 10.1038/nature14431 PMC4469479

[b11] Barker E. L., Moore K. R., Rakhshan F. & Blakely R. D. Transmembrane domain I contributes to the permeation pathway for serotonin and ions in the serotonin transporter. J. Neurosci. 19, 4705–4717 (1999). 10366604 10.1523/JNEUROSCI.19-12-04705.1999 PMC6782662

[b12] Henry L. K. . Tyr-95 and Ile-172 in transmembrane segments 1 and 3 of human serotonin transporters interact to establish high affinity recognition of antidepressants. J. Biol. Chem. 281, 2012–2023 (2006). 16272152 10.1074/jbc.M505055200

[b13] Andersen J. . Location of the antidepressant binding site in the serotonin transporter: importance of Ser-438 in recognition of citalopram and tricyclic antidepressants. J. Biol. Chem. 284, 10276–10284 (2009). 19213730 10.1074/jbc.M806907200 PMC2665081

[b14] Andersen J. . Mutational mapping and modeling of the binding site for (S)-citalopram in the human serotonin transporter. J. Biol. Chem. 285, 2051–2063 (2010). 19892699 10.1074/jbc.M109.072587 PMC2804362

[b15] Koldsø H. . The two enantiomers of citalopram bind to the human serotonin transporter in reversed orientations. J. Am. Chem. Soc. 132, 1311–1322 (2010). 20055463 10.1021/ja906923j

[b16] Sørensen L. . Interaction of antidepressants with the serotonin and norepinephrine transporters: mutational studies of the S1 substrate binding pocket. J. Biol. Chem. 287, 43694–43707 (2012). 23086945 10.1074/jbc.M112.342212 PMC3527955

[b17] Zhou Z. . LeuT-desipramine structure reveals how antidepressants block neurotransmitter reuptake. Science 317, 1390–1393 (2007). 17690258 10.1126/science.1147614 PMC3711652

[b18] Zhou Z. . Antidepressant specificity of serotonin transporter suggested by three LeuT-SSRI structures. Nat. Struct. Mol. Biol. 16, 652–657 (2009). 19430461 10.1038/nsmb.1602 PMC2758934

[b19] Singh S. K., Yamashita A. & Gouaux E. Antidepressant binding site in a bacterial homologue of neurotransmitter transporters. Nature 448, 952–956 (2007). 17687333 10.1038/nature06038

[b20] Singh S. K., Piscitelli C. L., Yamashita A. & Gouaux E. A competitive inhibitor traps LeuT in an open-to-out conformation. Science 322, 1655–1661 (2008). 19074341 10.1126/science.1166777 PMC2832577

[b21] Shi L., Quick M., Zhao Y., Weinstein H. & Javitch J. A. The mechanism of a neurotransmitter:sodium symporter-inward release of Na^+^ and substrate is triggered by substrate in a second binding site. Mol. Cell 30, 667–677 (2008). 18570870 10.1016/j.molcel.2008.05.008 PMC2826427

[b22] Plenge P. . Steric hindrance mutagenesis in the conserved extracellular vestibule impedes allosteric binding of antidepressants to the serotonin transporter. J. Biol. Chem. 287, 39316–39326 (2012). 23007398 10.1074/jbc.M112.371765 PMC3501018

[b23] Wang L., Xie J. & Schultz P. G. Expanding the genetic code. Annu. Rev. Biophys. Biomol. Struct. 35, 225–249 (2006). 16689635 10.1146/annurev.biophys.35.101105.121507

[b24] Liu C. C. & Schultz P. G. Adding new chemistries to the genetic code. Annu. Rev. Biochem. 79, 413–444 (2010). 20307192 10.1146/annurev.biochem.052308.105824

[b25] Davis L. & Chin J. W. Designer proteins: applications of genetic code expansion in cell biology. Nat. Rev. Mol. Cell Biol. 13, 168–182 (2012). 22334143 10.1038/nrm3286

[b26] Wang L., Brock A., Herberich B. & Schultz P. G. Expanding the genetic code of *Escherichia coli*. Science 292, 498–500 (2001). 11313494 10.1126/science.1060077

[b27] Mori H. & Ito K. Different modes of SecY-SecA interactions revealed by site-directed in vivo photo-cross-linking. Proc. Natl Acad. Sci. USA 103, 16159–16164 (2006). 17060619 10.1073/pnas.0606390103 PMC1621050

[b28] Hino N. . Protein photo-cross-linking in mammalian cells by site-specific incorporation of a photoreactive amino acid. Nat. Methods 2, 201–206 (2005). 15782189 10.1038/nmeth739

[b29] Chin J. W., Martin A. B., King D. S., Wang L. & Schultz P. G. Addition of a photocrosslinking amino acid to the genetic code of *Escherichia coli*. Proc. Natl Acad. Sci. USA 99, 11020–11024 (2002). 12154230 10.1073/pnas.172226299 PMC123203

[b30] Takimoto J. K., Adams K. L., Xiang Z. & Wang L. Improving orthogonal tRNA-synthetase recognition for efficient unnatural amino acid incorporation and application in mammalian cells. Mol. Biosyst. 5, 931–934 (2009). 19668857 10.1039/b904228h

[b31] Grunbeck A. . Genetically encoded photo-cross-linkers map the binding site of an allosteric drug on a G protein-coupled receptor. ACS Chem. Biol. 7, 967–972 (2012). 22455376 10.1021/cb300059z

[b32] Grunbeck A., Huber T., Sachdev P. & Sakmar T. P. Mapping the ligand-binding site on a G protein-coupled receptor (GPCR) using genetically encoded photocrosslinkers. Biochemistry 50, 3411–3413 (2011). 21417335 10.1021/bi200214r PMC3099303

[b33] Valentin-Hansen L. . Mapping substance P binding sites on the neurokinin-1 receptor using genetic incorporation of a photoreactive amino Acid. J. Biol. Chem. 289, 18045–18054 (2014). 24831006 10.1074/jbc.M113.527085 PMC4140293

[b34] Coin I., Perrin M. H., Vale W. W. & Wang L. Photo-cross-linkers incorporated into G-protein-coupled receptors in mammalian cells: a ligand comparison. Angew. Chem. Int. Ed. 50, 8077–8081 (2011). 10.1002/anie.201102646 PMC3262126 21751313

[b35] Ye S. . Site-specific incorporation of keto amino acids into functional G protein-coupled receptors using unnatural amino acid mutagenesis. J. Biol. Chem. 283, 1525–1533 (2008). 17993461 10.1074/jbc.M707355200

[b36] Ye S., Huber T., Vogel R. & Sakmar T. P. FTIR analysis of GPCR activation using azido probes. Nat. Chem. Biol. 5, 397–399 (2009). 19396177 10.1038/nchembio.167 PMC2875874

[b37] Ye S. . Tracking G-protein-coupled receptor activation using genetically encoded infrared probes. Nature 464, 1386–1389 (2010). 20383122 10.1038/nature08948

[b38] Coin I. . Genetically encoded chemical probes in cells reveal the binding path of urocortin-I to CRF class B GPCR. Cell 155, 1258–1269 (2013). 24290358 10.1016/j.cell.2013.11.008 PMC3916339

[b39] Klippenstein V., Ghisi V., Wietstruk M. & Plested A. J. Photoinactivation of glutamate receptors by genetically encoded unnatural amino acids. J. Neurosci. 34, 980–991 (2014). 24431456 10.1523/JNEUROSCI.3725-13.2014 PMC6608342

[b40] Zhu S. . Genetically encoding a light switch in an ionotropic glutamate receptor reveals subunit-specific interfaces. Proc. Natl Acad. Sci. USA 111, 6081–6086 (2014). 24715733 10.1073/pnas.1318808111 PMC4000820

[b41] Ye S., Riou M., Carvalho S. & Paoletti P. Expanding the genetic code in *Xenopus laevis* oocytes. Chembiochem. 14, 230–235 (2013). 23292655 10.1002/cbic.201200515

[b42] Andersen J. . Binding of the multimodal antidepressant drug vortioxetine to the human serotonin transporter. ACS Chem. Neurosci. 6, 1892–1900 (2015). 26389667 10.1021/acschemneuro.5b00225

[b43] Koban F. . A salt bridge linking the first intracellular loop with the C terminus facilitates the folding of the serotonin transporter. J. Biol. Chem. 290, 13263–13278 (2015). 25869136 10.1074/jbc.M115.641357 PMC4505579

[b44] Mørk A. . Pharmacological effects of Lu AA21004: a novel multimodal compound for the treatment of major depressive disorder. J. Pharmacol. Exp. Ther. 340, 666–675 (2012). 22171087 10.1124/jpet.111.189068

[b45] Bang-Andersen B. . Discovery of 1-[2-(2,4-dimethylphenylsulfanyl)phenyl]piperazine (Lu AA21004): a novel multimodal compound for the treatment of major depressive disorder. J. Med. Chem. 54, 3206–3221 (2011). 21486038 10.1021/jm101459g

[b46] Talvenheimo J., Fishkes H., Nelson P. J. & Rudnick G. The serotonin transporter-imipramine "receptor". J. Biol. Chem. 258, 6115–6119 (1983). 6853478

[b47] Kumar V. . Novel azido-iodo photoaffinity ligands for the human serotonin transporter based on the selective serotonin reuptake inhibitor (S)-citalopram. J. Med. Chem. 58, 5609–5619 (2015). 26153715 10.1021/acs.jmedchem.5b00682 PMC4515784

[b48] Dahal R. A. . Computational and biochemical docking of the irreversible cocaine analog RTI 82 directly demonstrates ligand positioning in the dopamine transporter central substrate-binding site. J. Biol. Chem. 289, 29712–29727 (2014). 25179220 10.1074/jbc.M114.571521 PMC4207985

[b49] Parnas M. L. . Labeling of dopamine transporter transmembrane domain 1 with the tropane ligand *N*-[4-(4-azido-3-[^125^I]iodophenyl)butyl]-2*β*-carbomethoxy-3*β*-(4-chlorophenyl) tropane implicates proximity of cocaine and substrate active sites. Mol. Pharmacol. 73, 1141–1150 (2008). 18216182 10.1124/mol.107.043679

[b50] Vaughan R. A., Agoston G. E., Lever J. R. & Newman A. H. Differential binding of tropane-based photoaffinity ligands on the dopamine transporter. J. Neurosci. 19, 630–636 (1999). 9880583 10.1523/JNEUROSCI.19-02-00630.1999 PMC6782203

[b51] Sinning S. . Binding and orientation of tricyclic antidepressants within the central substrate site of the human serotonin transporter. J. Biol. Chem. 285, 8363–8374 (2010). 19948720 10.1074/jbc.M109.045401 PMC2832986

[b52] Sarker S. . The high-affinity binding site for tricyclic antidepressants resides in the outer vestibule of the serotonin transporter. Mol. Pharmacol. 78, 1026–1035 (2010). 20829432 10.1124/mol.110.067538 PMC4513247

[b53] Andersen J. . Molecular basis for selective serotonin reuptake inhibition by the antidepressant agent fluoxetine (Prozac). Mol. Pharmacol. 85, 703–714 (2014). 24516100 10.1124/mol.113.091249

[b54] Huber T. & Sakmar T. P. Chemical biology methods for investigating G protein-coupled receptor signaling. Chem. Biol. 21, 1224–1237 (2014). 25237865 10.1016/j.chembiol.2014.08.009

[b55] Cheng Y. & Prusoff W. H. Relationship between the inhibition constant (K_I_) and the concentration of inhibitor which causes 50 per cent inhibition (I_50_) of an enzymatic reaction. Biochem. Pharmacol. 22, 3099–3108 (1973). 4202581 10.1016/0006-2952(73)90196-2

