# Supplementary material for: Genetically encoded photocrosslinkers locate the high-affinity binding site of antidepressant drugs in the human serotonin transporter
Source: Nat Commun. 2016 Apr 19;7:11261. doi: 10.1038/ncomms11261 (PMC4838859; doi:10.1038/ncomms11261)
Supplement: Supplementary Information — Supplementary Figures 1-6, Supplementary Tables 1-3 and Supplementary References, [file ncomms11261-s1.pdf]

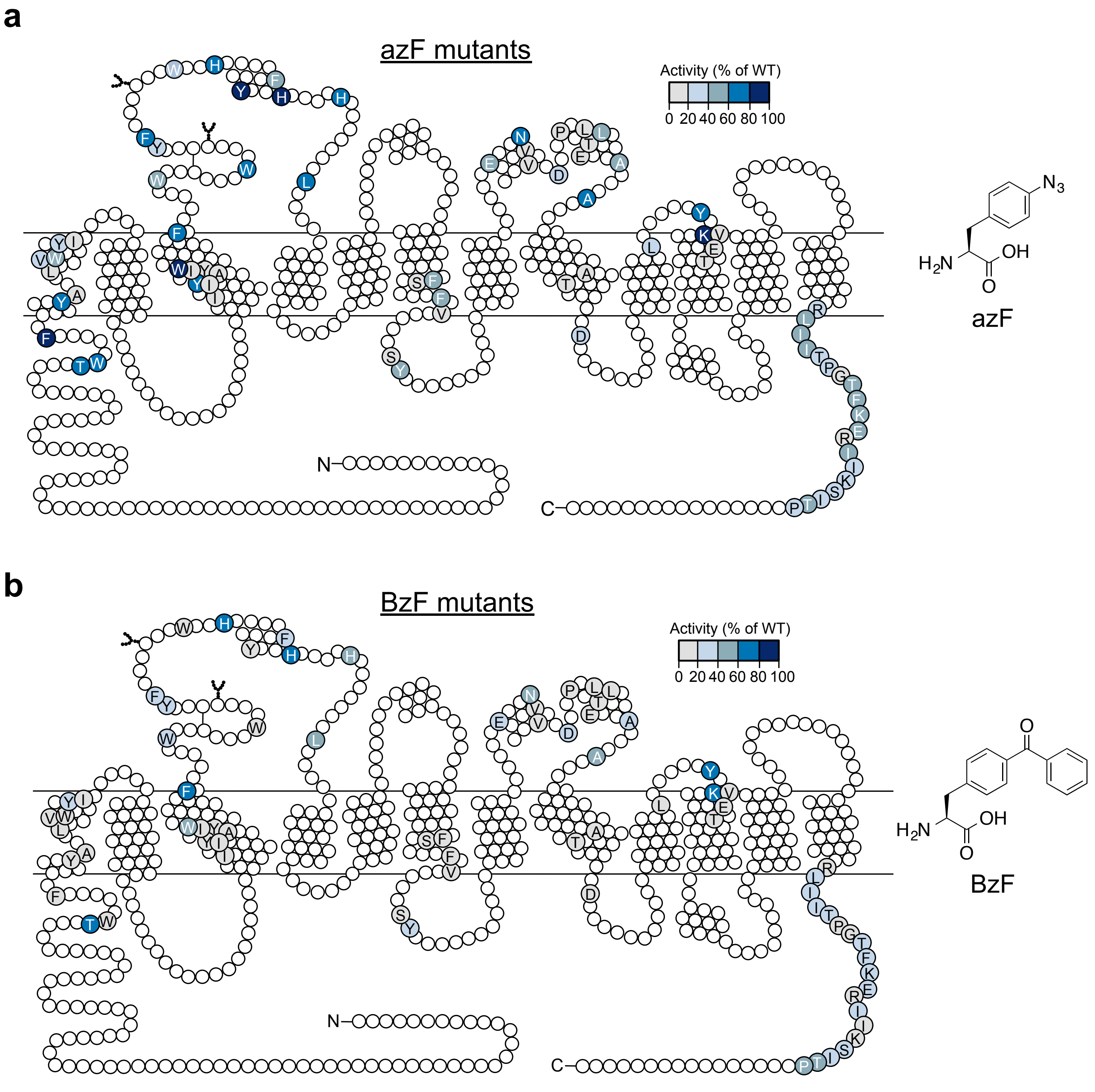

Supplementary Figure 1: Functional activity of azF and BzF mutants. Location of azF (**a**) and BzF (**b**) mutants plotted onto the 2D topology of human SERT, and color-coded according to their functional activity relative to WT SERT (see **Supplementary Table 1**). The chemical structure of azF and BzF are shown on the right.

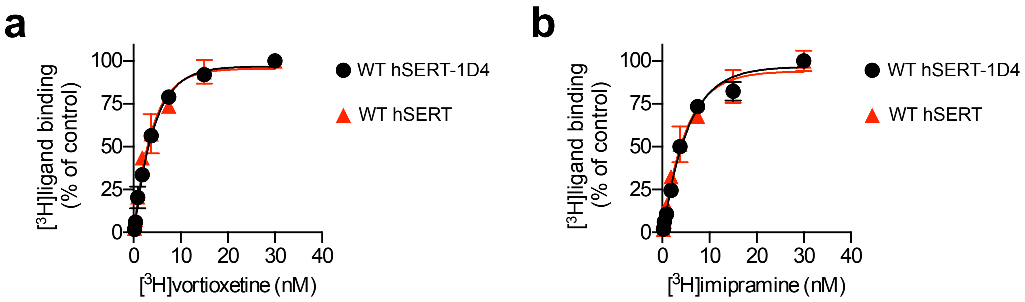

Supplementary Figure 2: Effect of 1D4 tag on ligand binding. Saturation binding curves for [^{3}H]vortioxetine (**a**) and [^{3}H]imipramine (**b**) at WT hSERT and hSERT fused to a 1D4 tag at the C-terminal. The 1D4 tag did not affect the binding affinity (K_d) of hSERT to vortoxetine (6.1 ± 1.1 nM and 6.3 ± 0.7 nM for WT and 1D4-tagged, respectively) or imipramine (4.5 ± 0.7 nM and 7.2 ± 0.8 nM for WT and 1D4 tagged, respectively).

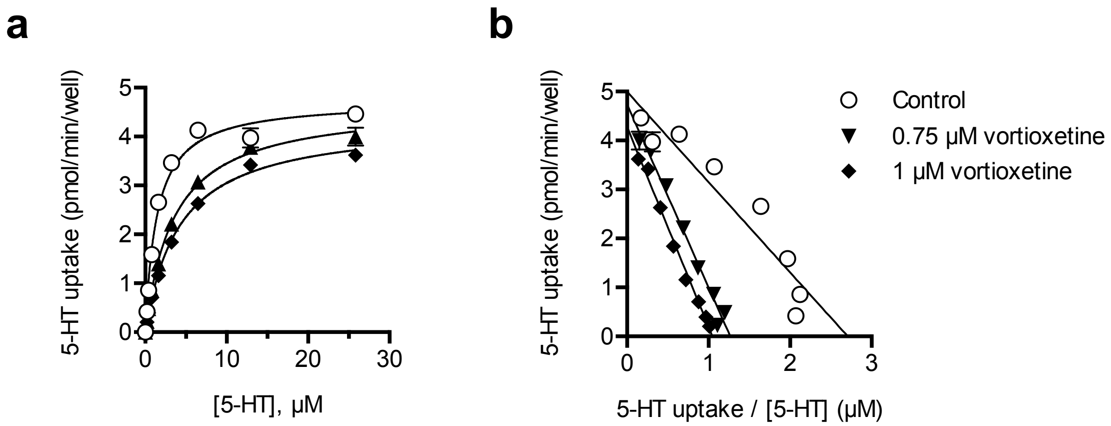

Supplementary Figure 3: Competitive inhibition of 5-HT transport by vortioxetine. Substrate K_m and V_{max} values for 5-HT uptake into hSERT WT were determined in [^{3}H]5-HT saturation uptake assays as described in "Methods". In paired experiments, the saturation uptake assays were carried out in the absence (control) or in the presence of a fixed concentration (0.75 or 1 μM) of vortioxetine. To ensure that 5-HT and vortioxetine had equal access to their binding sites on hSERT, they were added simultaneously in these experiments. (**a**) 5-HT uptake curves from a representative saturation uptake experiment. Error bars represent s.e.m. and are shown when larger than symbols. The same data are shown in the form of an Eadie-Hofstee plot in (**b**). The K_m values (in μM) were 1.67 ± 0.12 for control, 3.36 ± 0.23 for 0.75 μM vortioxetine and 4.68 ± 0.28 for 1 μM vortioxetine. K_m values for 0.75 and 1 μM vortioxetine were significantly different from control ($p < 0.05$; paired Student's *t*-test). V_{max} values (in pmol min^{-1} per well) were 4.21 ± 0.45 for control, 4.19 ± 0.50 for 0.75 μM vortioxetine and 4.03 ± 0.36 for 1 μM vortioxetine. V_{max} values for 0.75 and 1 μM vortioxetine were not significantly different from control ($p > 0.05$; paired Student's *t*-test). K_m and V_{max} values are mean ± s.e.m. from five experiments each performed in triplicate.

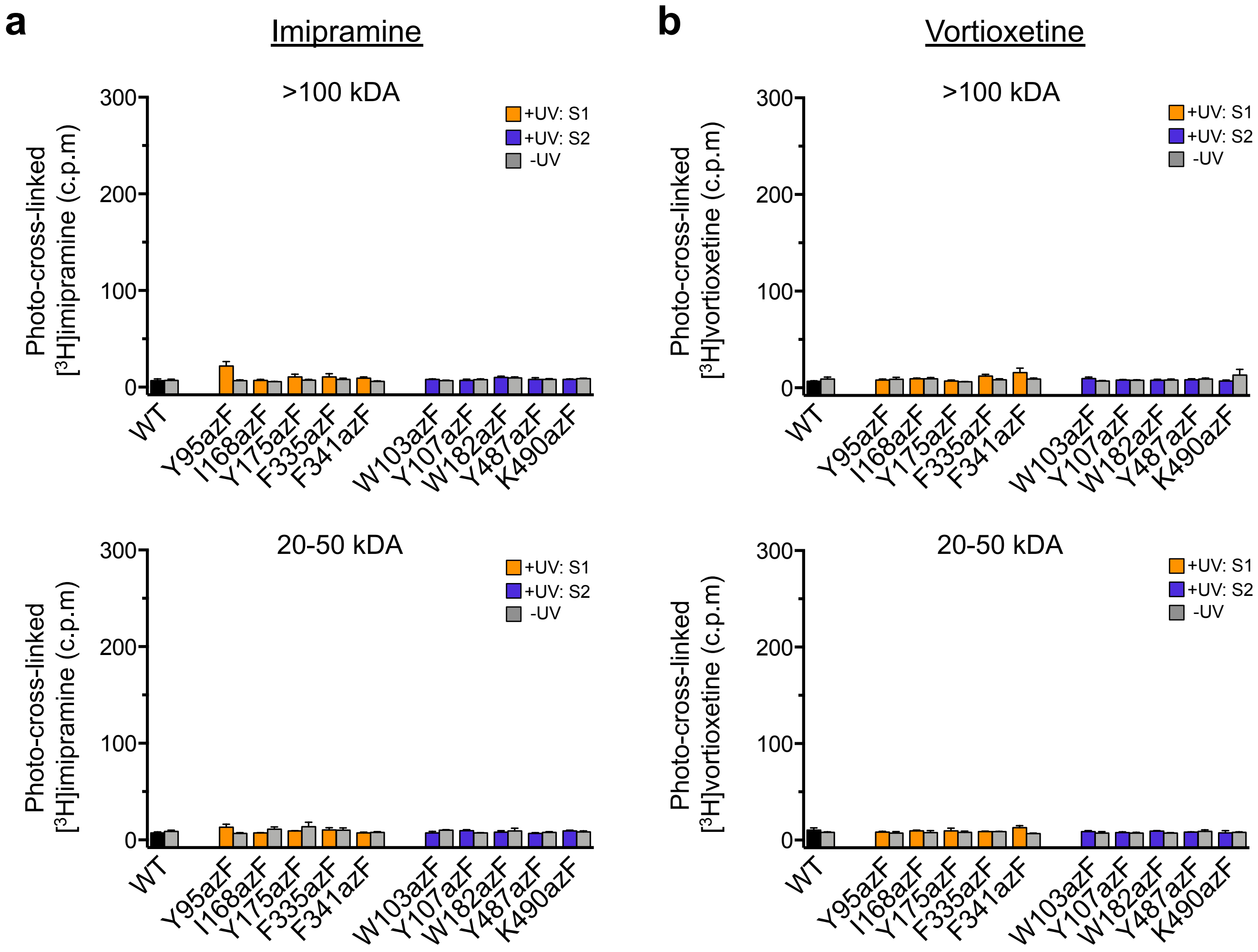

Supplementary Figure 4: Photo-cross-linking of imipramine and vortioxetine using azF. Quantification of radioactivity (c.p.m) on PVDF membrane segments containing protein with the molecular weight of >100 kDa (upper) or 20-50 kDa (lower) from [^{3}H]imipramine (**a**) and [^{3}H]vortioxetine (**b**) photo-cross-linking experiments. A radioactive signal indicates a positive cross-link between azF in hSERT and the radiolabelled inhibitor. Data are represented as mean ± s.e.m from 3-7 independent experiments.

a

b

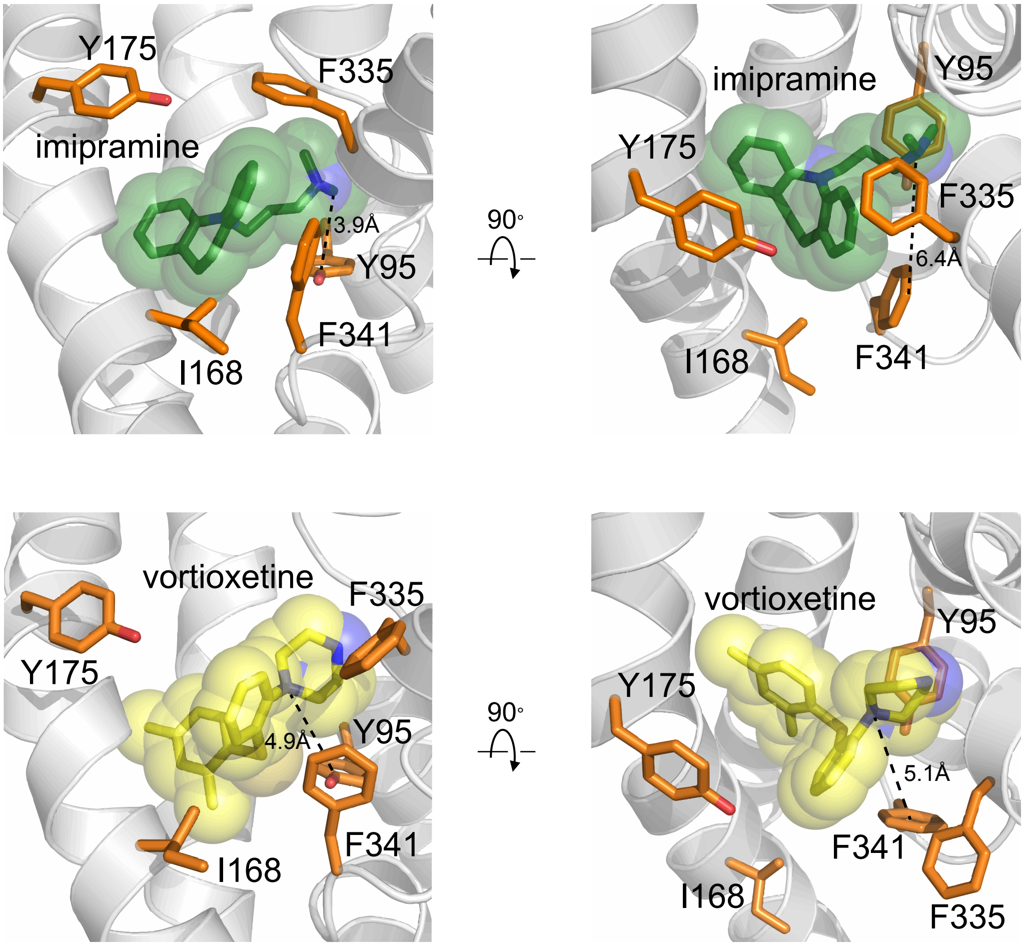

Supplementary Figure 5: Docking models of imipramine and vortioxetine in hSERT. Docking models of imipramine[1] (**a**) and vortioxetine[2] (**b**) binding in the S1 site in hSERT (same models as shown in **Fig. 4d** and **Fig. 4e**, respectively). Imipramine is shown in green (**a**), vortioxetine is shown in yellow (**b**) and the five S1 residues that were mutated to azF are shown as orange sticks. The models are shown from within the plane of the membrane (*left*) and from the extracellular side of the membrane (*right*). Distances between the dimethylamino moiety of imipramine and the piperazine ring of vortioxetine to Y95 (*left panels*) and F341 (*right panels*) are indicated on the figure.

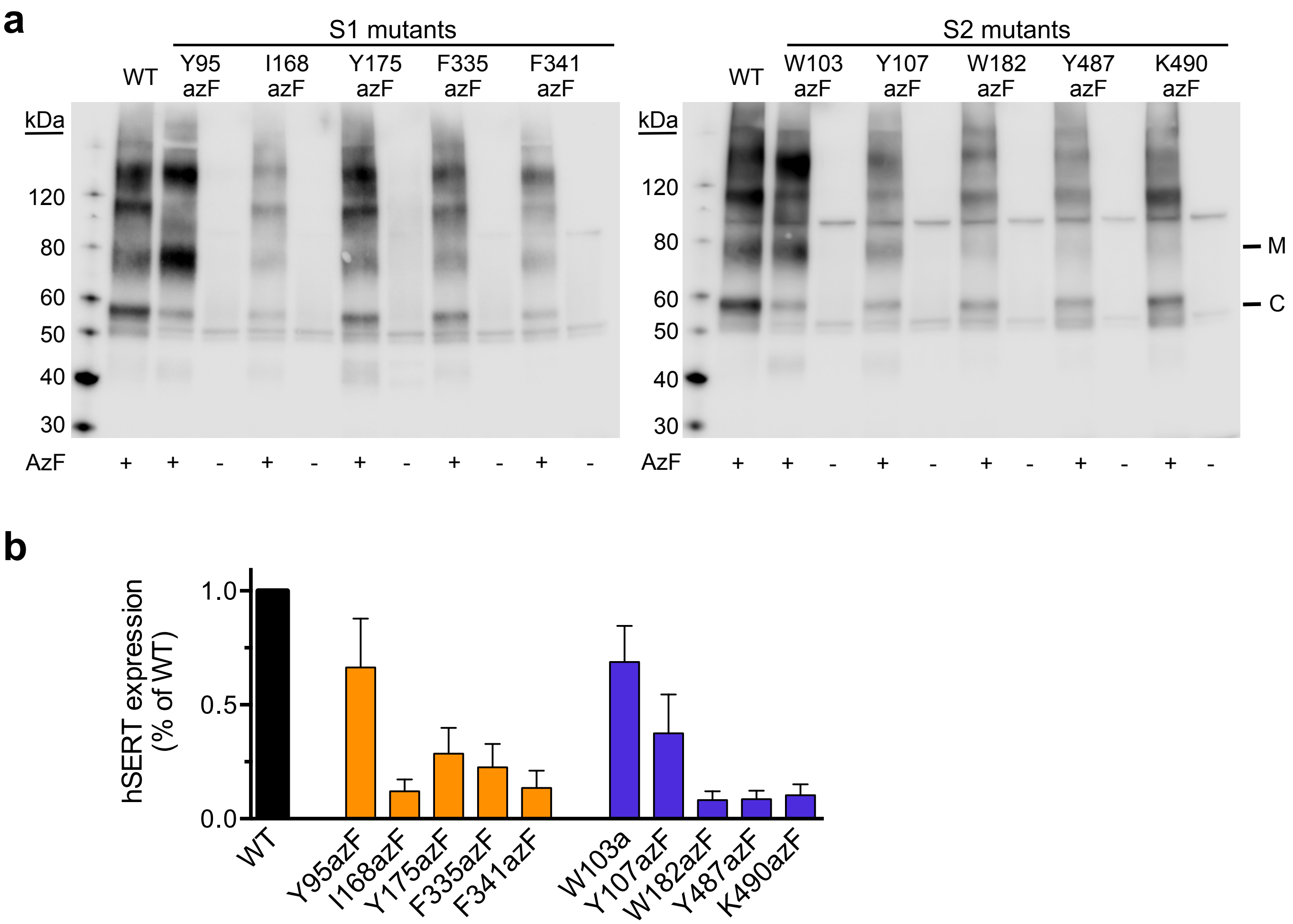

Supplementary Figure 6: Expression level of hSERT azF mutants. (a) Immunoblot analysis of immunoprecipitated lysates from HEK293T cells expressing WT hSERT or azF mutants in the presence or absence of azF, using a mAb against a C-terminal 1D4 epitope fused to hSERT (as described in "Methods"). C, core glycosylated hSERT protein; M, mature glycosylated hSERT protein[3]. **(b)** Expression levels of hSERT azF mutants (in the presence of azF) relative to hSERT WT. Bars represent mean ± s.e.m. of quantified expression levels of mature glycosylated hSERT protein from 5-6 independent blots. Bands were quantified with GelQuant software (DNR Bio-Imaging Systems Ltd.)

Region	Position	Transport activity azF	Transport activity BzF	Region	Position	Transport activity azF	Transport activity BzF
		% of WT	*% of WT*			*% of WT*	*% of WT*
N-terminus	T81	75 ± 2	76 ± 3				
N-terminus	W82[a]	76 ± 3	15 ± 1	EL4	V397[b]	NF	NF
N-terminus	F88[a]	82 ± 5	NF	🔵 EL4	D400	34 ± 2	32 ± 2
🟠 TM1	Y95	66 ± 1	NF	EL4	P403[b]	NF	NF
🟠 TM1	A96	NF	NF	🔵 EL4	L405	NF	NF
🟠 TM1	L99	NF	NF	🔵 EL4	L406[a]	49 ± 4	NF
TM1	V102	24 ± 2	NF	EL4	T409[b]	NF	NF
🔵 TM1	W103	71 ± 2	16 ± 1	EL4	E412[b]	14 ± 3	6 ± 3
🔵 TM1	Y107	21 ± 3	20 ± 2	EL4	A415[b]	54 ± 4	22 ± 1
🔵 TM1	I108	NF	NF	EL4	A419[a]	62 ± 2	48 ± 8
🟠 TM3	I168	16 ± 1	NF	🟠 TM8	T439	NF	NF
🟠 TM3	I172	NF	NF	🟠 TM8	A441	NF	NF
🟠 TM3	A173	NF	NF	IL4	D452[a]	27 ± 3	NF
🟠 TM3	Y175	69 ± 6	15 ± 2	TM9	L481[a]	57 ± 1	13 ± 5
🟠 TM3	Y176	NF	NF	EL5	Y487	80 ± 5	62 ± 5
🔵 TM3	I179	NF	NF	🔵 TM10	V489[a]	NF	NF
🔵 TM3	W182	85 ± 7	49 ± 3	🔵 TM10	K490	82 ± 4	60 ± 1
TM3	F191	77 ± 4	67 ± 7	🔵 TM10	E493[a]	NF	NF
EL2	W197	56 ± 4	29 ± 4	🟠 TM10	T497	NF	NF
EL2	W204	74 ± 3	11 ± 1	C-terminus	R596	29 ± 2	13 ± 2
EL2	Y212	37 ± 4	26 ± 4	C-terminus	L597	46 ± 4	26 ± 3
EL2	F213	66 ± 2	39 ± 5	C-terminus	I598	43 ± 8	21 ± 7
EL2	W220	48 ± 4	11 ± 1	C-terminus	I599	43 ± 7	29 ± 5
EL2	H223	78 ± 3	67 ± 6	C-terminus	T600	37 ± 5	21 ± 5
EL2	F231	59 ± 3	28 ± 8	C-terminus	P601	21 ± 6	13 ± 4
EL2	Y232	82 ± 2	11 ± 2	C-terminus	G602	6 ± 2	8 ± 1
EL2	H235	83 ± 3	62 ± 3	C-terminus	T603	42 ± 6	29 ± 6
EL2	H240	68 ± 4	58 ± 5	C-terminus	F604	47 ± 7	31 ± 5
EL2	L248	70 ± 3	59 ± 3	C-terminus	K605	47 ± 5	29 ± 6
🟠 TM6	F335	50 ± 5	NF	C-terminus	E606	47 ± 1	39 ± 2
🟠 TM6	S336	NF	NF	C-terminus	R607	15 ± 5	8 ± 1
🟠 TM6	F341	56 ± 3	NF	C-terminus	I608	46 ± 4	30 ± 5
🟠 TM6	V343	NF	NF	C-terminus	I609	32 ± 3	17 ± 3
TM6	S349[a]	NF	NF	C-terminus	K610	27 ± 1	18 ± 3
IL3	Y350[a]	51 ± 4	20 ± 4	C-terminus	S611	29 ± 4	23 ± 5
EL4	E388[b]	51 ± 7	37 ± 5	C-terminus	I612	38 ± 3	28 ± 2
EL4	N391[b]	65 ± 15	51 ± 13	C-terminus	T613	49 ± 4	47 ± 1
EL4	V394[b]	NF	NF	C-terminus	P614	37 ± 3	49 ± 3

[a] C-terminally fused to GFP[2]. [b] C-terminally fused to 6 x His tag. 🟠 S1 site. 🔵 S2 site.

Supplementary Table 1. Transport activity of azF and BzF mutants. Transport activities were determined in a [^{3}H]5-HT uptake assay as described under "Methods". Unless otherwise noted, the transporter protein was C-terminally fused to a 1D4 tag. Results are presented as mean ± s.e.m. from two to six independent experiments each performed in triplicate. TM = transmembrane helix, EL = extracellular loop, NF = non-functional (≤ 5% of WT).

Residue type/location	Number of mutants	Functional mutants	
		azF	BzF
Total	75	55 (73%)	47 (63%)
Aromatic	23	22 (96%)	18 (78%)
Termini	22	22 (100%)	21 (95%)
Loop	28	22 (79%)	21 (75%)
TM	25	12 (44%)	5 (20%)

Supplementary Table 2. Transport activities of azF and BzF mutant according to residue type and location in hSERT.

	K_m	V_{max} [a]	K_i (vortioxetine)	K_i (imipramine)
	µM	*% of WT*	*nM*	*nM*
SERT WT	2.24 ± 0.25		202 ± 19	98 ± 16
**S1 mutants**				
Y95azF	1.17 ± 0.12*	36 ± 2	1245 ± 125*	2091 ± 120*
I168azF	0.44 ± 0.08*	2 ± 0.3	19 ± 3*	3 ± 0.6*
Y175azF	0.44 ± 0.08*	10 ± 1	53 ± 6*	12 ± 2*
F335azF	0.17 ± 0.03*	3 ± 0.4	54 ± 9*	34 ± 6*
F341azF	0.40 ± 0.07*	9 ± 0.8	43 ± 5*	79 ± 6
**S2 mutants**				
W103azF	1.47 ± 0.21*	38 ± 4	149 ± 12	55 ± 5
Y107azF	0.61 ± 0.13*	2 ± 0.5	38 ± 9*	8 ± 0.3*
W182azF	0.66 ± 0.10*	16 ± 1	74 ± 9*	17 ± 2*
Y487azF	0.48 ± 0.07*	10 ± 0.6	96 ± 16*	27 ± 3*
K490azF	0.18 ± 0.03*	5 ± 0.3	56 ± 20*	14 ± 3*

Supplementary Table 3. Impact of azF mutants on transport kinetics and inhibitory potency of vortioxetine and imipramine. Substrate K_m and V_{max} values and inhibitor K_i values were determined in [^{3}H]5-HT uptake assays as described in ”Methods”. Results are presented as mean ± s.e.m. from 3–11 independent experiments each performed in triplicate. *Asteriks* (*) denote significantly different K_m or K_i value compared to SERT WT ($p < 0.05$; Student’s *t*-test).

[a] V_{max} is expressed as percentage of SERT WT determined in parallel. For SERT WT, V_{max} (mean ± s.e.m.) was 4.09 ± 0.82 pmol min^{-1} per well (n = 11).

Supplementary References

1. Sinning, S. *et* al. Binding and orientation of tricyclic antidepressants within the central substrate site of the human serotonin transporter. *J. Biol. Chem.* **285,** 8363-8374 (2010).
2. Andersen, J. *et al.* Binding of the multimodal antidepressant drug vortioxetine to the human serotonin transporter. *ACS Chem. Neurosci.* **6,** 1892-1900 (2015).
3. Koban, F. et al. A salt bridge linking the first intracellular loop with the C terminus facilitates the folding of the serotonin transporter. J. Biol. Chem. 290, 13263-13278 (2015).
